# An Immunoenzymatic Method for the Determination of Ochratoxin A in Biological Liquids (Colostrum and Cow’s Milk)

**DOI:** 10.3390/toxins13100673

**Published:** 2021-09-22

**Authors:** Magdalena Cuciureanu, Cristina Tuchiluș, Anca Vartolomei, Bogdan Ionel Tamba, Lorena Filip

**Affiliations:** 1 Departament of Pharmacology, “Grigore T. Popa” University of Medicine and Pharmacy, 700115 Iasi, Romania; 2 Departament of Microbiology, “Grigore T. Popa” University of Medicine and Pharmacy, 700115 Iasi, Romania; cristina.tuchilus@umfiasi.ro; 3 Department of Environmental and Food Chemistry, “Grigore T. Popa” University of Medicine and Pharmacy, 700115 Iasi, Romania; anca_farma@yahoo.com; 4 Center for Advanced Research and Development in Experimental Medicine (CEMEX), “Grigore T. Popa” University of Medicine and Pharmacy, 700115 Iasi, Romania; bogdan.tamba@umfiasi.ro; 5 Departament of Bromatology, Hygiene, Nutrition, “Iuliu Hatieganu” University of Medicine and Pharmacy, 400012 Cluj Napoca, Romania; lfilip@umfcluj.ro

**Keywords:** ochratoxin A, enzyme immunoassay, milk

## Abstract

Ochratoxins are mycotoxins that have been extensively studied lately due to the multiple toxic effects such as nephrotoxicity, hepatotoxicity, and carcinogenicity. These toxins contaminate plant and animal foods and after ingestion they reach into body fluids. The method of competitive direct enzyme immunoassay, in the solid phase, was validated through the determination of specific parameters (performance, linearity, recovery percentage, limit of detection, limit of quantification). The validated method was used to determine ochratoxin A in colostrum and cow’s milk. The method applied for the determination of ochratoxin A was linear for the concentration range of 0.0–0.5 ng/mL, the value for the regression coefficient (r) was 0.9838. Ochratoxin A was present in 91.67% of the colostrum and in 93.33% of cow’s milk samples. The linearity of the method, demonstrated for very low concentrations of analyte, the detection limit as well as the limit of quantification recommend the method for the determinations of micro-pollutants from foods, including biological fluids.

## 1. Introduction

**Figure 1:**
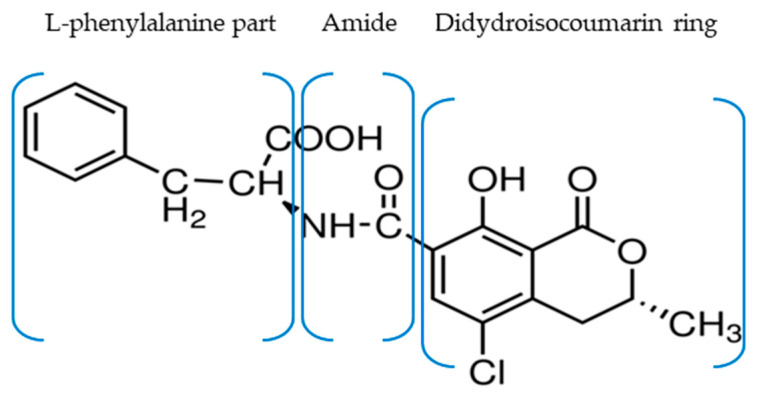
OTA structure containing a dihydroisocoumarin ring that is amide-linked to L-phenylalanine.

Mycotoxins—namely aflatoxins, ochratoxins, patulin, fumonisins, zearalenone, trichothecenes, and ergot alkaloids are secondary metabolites of certain fungi that contaminate different types of crops (before or after harvest) depending on temperature, humidity, pH, and oxygen [1,2,3]. There are several types of ochratoxins (OTA, OTB, OTC, and OTα) but OTA is the most studied one due to its deleterious effects on health. Chemically characterized in 1965, ochratoxin A (OTA) is a mycotoxin that consists of a para-chlorophenolic moiety containing a dihydroisocoumarin group that is amide-linked to L-phenylalanine) (Figure 1) [1,2].

OTA is produced by three major species of fungi *Aspergillus carbonarius*, *Penicillium verrucosum,* and *Aspergillus ochraceus* but can also be produced by *P. nordicum*, *A. niger*, *A. tubingensis*, and others [2,4]. *Penicillium verrucosum* produces OTA in cereals from cold areas, *A. carbonarius* in grapes, red pepper, and coffee beans and *A. ochraceus* in soya beans, cereals, and nuts in warm and tropical climates [2,3,5]. 

Moreover, human exposure to mycotoxins can also occur through the ingestion of dairy products and meat obtained from animals fed with contaminated food [1,2]. Particular concerns must be raised regarding the presence of these compounds in breast, cow, or formula milk as increased OTA concentrations are associated with infant growth faltering [6]. Infants are more susceptible to the harmful effects of mycotoxins due to immature elimination pathways, incomplete organism development, elevated intake/body weight ratio, and high metabolic rate [3,5]. Some authors consider that the negative consequences on infants are three times more amplified than in adults [5]. OTA’s negative effects are even more dangerous due to its synergic activity with other mycotoxins [7].

In 1993, based on demonstrated carcinogenicity in animal studies, IARC (International Agency for Research on Cancer) identified OTA as a possible human carcinogen and included this mycotoxin in group 2B [8]. OTA toxicity is presumably due to its direct genotoxic effect through DNA binding and/or epigenetic or non-genotoxic mechanisms [1,9]. Pharmacokinetic characteristics such as long half-life time (35–70 days), high affinity for proteins (particularly serum albumin), and multiple active compounds obtained through OTA metabolism facilitate even more bio-accumulation and detrimental effects on human health [1,2,10,11]. Multiple data show that, in many species, high OTA levels are associated with nephrotoxic, hepatotoxic, teratogenic, immunotoxic, hematotoxic, neurotoxic effects, and reproductive adverse effects [1,12,13]. Changes in oestradiol, thyroxin, testosterone, insulin, prolactin, and cortisol levels were also described [11,14,15].

Cell cultures experiments demonstrated that OTA increased apoptosis-related indices, mitochondrial dysfunction, mitochondria-dependent apoptosis activation, and interfered with cytokine pathways [9,16]. In HK-2 cells, OTA induced apoptosis through regulation of the PTEN/AKT signaling pathway via disrupting lipid raft formation [17]. OTA exacerbates oxidative stress and raises 8-hydroxydeoxyguanosine (8-OHdG) levels in DNA which links increased production of reactive oxygen species with genotoxic effects. Still, the direct production of specific OTA-induced DNA adducts is still debatable [11]. Even though there is a possible connection with bladder or hepatocellular cancer in humans, to date, epidemiological studies have not provided feasible proof [11]. 

It is considered that OTA accelerates the progression of some severe neurological pathologies such as Alzheimer’s and Parkinson’s disease [9,18]. Activation of different mechanisms such as p38 MAPK, JNKs, and ERKs dysfunctions, BDNF disruption, TH overexpression, caspase-3 and -9 activation, and ERK-1/2 phosphorylation is connected with Alzheimer’s disease evolution whereas, in Parkinson’s cases, impairment of chaperone-mediated autophagy and diminished alpha-synuclein turnover due to miRNAs dysfunction is described [9,18].

Similarly, OTA-induced neurotoxic effects were proven in glial and astrocyte cell lines. In NHA-SV40LT cells, OTA induced G0/G1 cell cycle arrest and increased cytosolic and mitochondrial calcium levels with subsequent elevation of MMP2 and PLAUR mRNA expression [19]. Interestingly, in murine studies, low doses of OTA produced changes in gut microbiota with a reduction in fecal microbiota variety and boost of *Bacterioides* at the phylum level [20]. In the absence of the adequate ruminal microbiota for the transformation of OTA to less injurious compounds, monogastric species, such as humans, are at higher risk [21]. Given the gut–brain connection, maintaining the microbiota balance plays an important role in wellness. In a logical manner, different microorganisms mixture from kefir can be exploited in order to reverse the side-effects generated by OTA. Taheur et al. (2017) indicated that *Lactobacillus kefiri*, *Kazachstania servazzii*, and *Acetobacter syzygii* can reduce the gastrointestinal absorption of milk-cultivated OTA by 82–100% [22]. Other strategies such as curcumin or tryptophan supplementation and essential oils administration were suggested to lower OTA’s impact [1,23,24]. 

The new research illustrates that OTA increases susceptibility for autoimmune diseases. In a collagen-induced arthritis model, OTA acted as a trigger for rheumatoid arthritis by stimulating the differentiation of naïve T cells into Th1 cells and IL17 production via Stat signaling pathways [25]. In addition, OTA exhibits diabetogenic effects by exerting injurious responses in Langerhans islets. In addition, decreasing insulin levels also raised glucagon expression [26].

The clinical manifestations of OTA intoxication (ochratoxicoses) are similar in most affected species; however, animals differ by their sensitivity to mycotoxins. Ochratoxicoses have been confirmed in humans in the Balkan region since 1994. The nephrotoxic effect of OTA in humans was associated with the development of Balkan endemic nephropathy (NEB) [27,28,29,30]. NEB was diagnosed in Romania, Bulgaria, and Yugoslavia. In Romania, five centers of the outbreak were identified in Oltenia and one in Banat [31]. Furthermore, exposure to OTA was correlated with the incidence of chronic interstitial nephropathy in the Maghreb region [2,32] and nephritic syndrome in Egypt [33,34]. Interestingly, elevated levels of OTA identified in infants (via breastfeeding) were related to increased proteinuria, and the presence of OTA in human breast milk enhanced the risk of HIV transmission (by lowering TNF alpha levels and elevating C-X-C motif chemokine 10 concentration) [6]. Another worrisome problem is the existing link between mycotoxins levels in amniotic fluid samples and genetic defects in fetuses as indicated by Gromadzka et al., 2020 [35].

In this context, the aim of this paper is to standardize an immunoenzymatic method for the detection of OTA and to detect this mycotoxin’s concentration in breast milk from first the three days (colostrum) and cow’s milk. 

## 2. Results

### 2.1. Validation of the Direct, Competitive Immunoenzymatic Method 

The validation parameters of the method (performance, linearity, reproducibility, recovery, limit of detection, limit of quantification) were established through its application on colostrum and cow’s milk. 

#### 2.1.1. Performance of the Method 

The absorbance of (A) calibrators and samples were expressed as a percentage from the linking absorbance mean (antigen–antibody) at zero inhibition (A_0_): %A_0_ = (As/A_0_) × 100, As = A standard or sample, A_0_ = mean of absorbance at zero inhibition.

**Figure 2:**
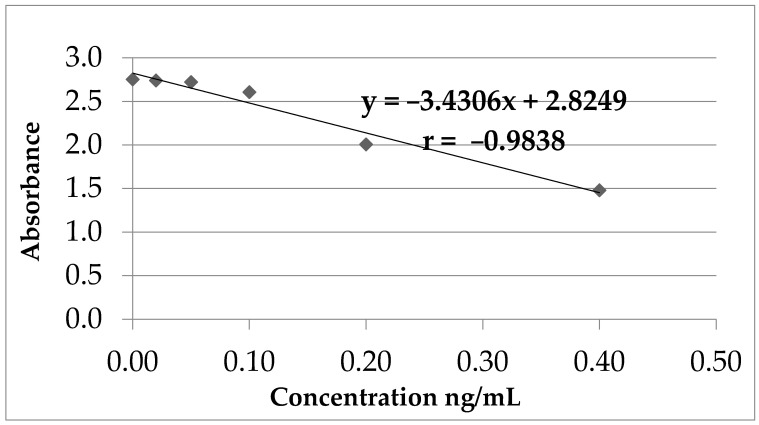
OTA calibration curve.

Based on the calculated values (%A_0_) for the standard concentrations (calibrators) and the concentration logarithm, a calibration curve was created (Figure 2). 

The two types of samples were measured and compared with zero concentration calibrator (blank) and with the lowest OTA concentration calibrator (other than zero) (Table 1). Twelve readings were made for each type of sample.

#### 2.1.2. Method Linearity (Calibration Curve)

The indirect proportional relationship between OTA concentration and absorbance (method linearity) was established for OTA concentrations between 0 and 0.4 ng/mL (Table 2, Figure 2).

The results obtained after statistical analysis of the calibration parameters corresponding to OTA were included in Table 3, Table 4 and Table 5.

#### 2.1.3. The Minimum Detection Limit (Milk)

Following the analysis of twenty milk samples negative for OTA, the minimum detection limit (DL) was calculated as the mean + 3 standard deviations (SD). The minimum detection limit (calculated as indicated above) represented the minimum concentration of the analyte that can be differentiated from the matrix effect with a certain level of trust and which can be distinguished from zero.

For the determination of the detection limit (Table 6) the following formula was used: DL = Mean (concentration) + 3 SD. 

#### 2.1.4. The Minimum Limit of Quantification (Milk)

The limit of quantification for milk samples (Table 6). The lowest concentration of analyte that can be determined with an acceptable level of repeatability and accuracy (CV < 20%) was calculated using the formula: LQ = Mean (concentration) + 10 SD.

#### 2.1.5. Recovery (Recovery Percentage)

The establishment of the recovery percentage was accomplished by enriching the milk samples with OTA −0.2 ng/mL; the samples were extracted and analyzed after 24 h of rest. The enrichment and withdrawal operations were repeated three times for each sample. The buffer washing solution PBS/Tween was enriched and extracted in the same way as the control samples (Table 7). 

The concentration of the PBS control solution was 0.214 ± 0.011 ng/mL and the quotient variation was 5.1% (*n* = 8).

The recovery percentages higher than 100% for cow’s milk samples indicated an intrinsic OTA level between 0.0 and 0.08 ng/mL. 

According to these values, the following data were obtained:

- equation of calibration line: *y* = −3.4306*x* + 2.8249
- standard error of regression line: ES = 0.1052

The method of analysis of OTA through the described protocol is linear on the concentration domain between 0.0–0.5 ng/mL, with a regression quotient (r) of 0.9838.

### 2.2. Determination of OTA in Human Milk Samples

**Figure 3:**
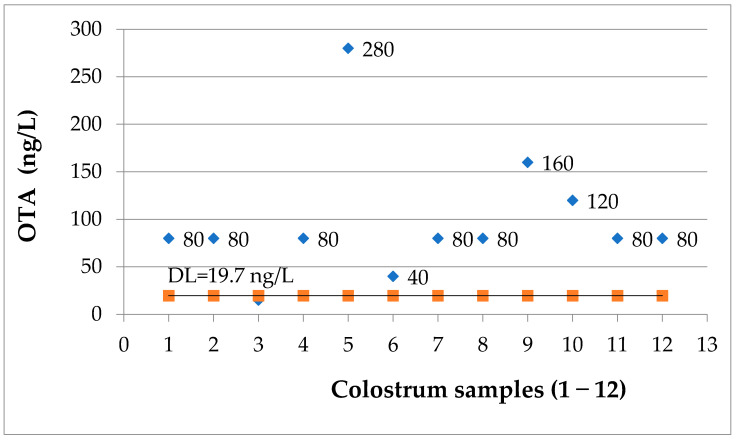
OTA levels (ng/L) in colostrum samples; DL = 19.7 ng/L.

91.67% percent of the 12 samples of colostrum were positive for OTA (Figure 3). For only one of the samples (9.09%), the OTA concentration was under the detection limit of the applied ELISA assay. The determined OTA concentration intervals are: less than 50 ng/L = one sample (9.09%); 51–100 ng/L = seven samples (63.64%); 101–200 ng/L = two samples (18.18%) and 201–300 ng/L = one sample (9.08%). 

### 2.3. Determination of OTA in Cow’s Milk Samples

**Figure 4:**
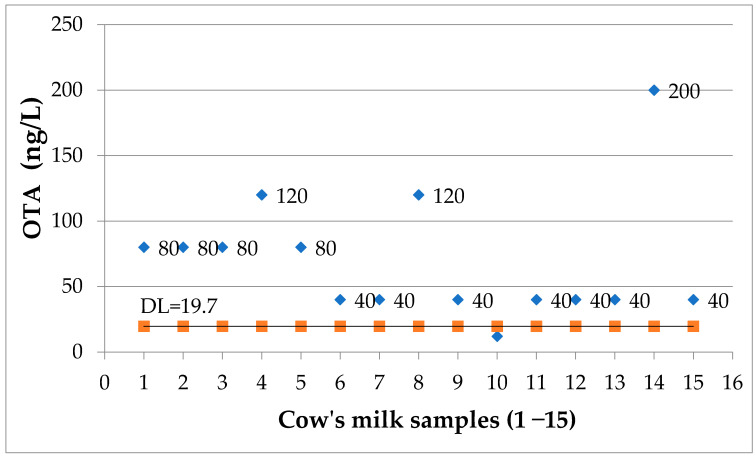
OTA levels (ng/L) in cow’s milk samples; DL = 19.7 ng/L.

93.33% percent of the 15 samples of cow’s milk were positive for OTA (Figure 4). For only one of the samples (6.66%), the OTA concentration was under the detection limit of the applied ELISA assay. The determined OTA concentration intervals are: less than 50 ng/L = seven samples (46.66%); 51–100 ng/L = four samples (26.66%); 101–200 ng/L = three samples (20%). No correlation with age, diet or mother’s place of residence could be extrapolated from our data (Tables S1 and S2).

## 3. Discussions

According to some authors, OTA concentrations in milk are 4–10 times lower than in blood which requires detection methods of high sensitivity [2,36]. Analytical methods for OTA detection include thin-layer chromatography (TLC), high-performance liquid chromatography (HPLC) with fluorescence detection (FLD), liquid chromatography (LC) coupled with a fluorometric detector for highly sensitive detection signal, LC-MS, LC-MS/MS, inductively coupled plasma mass spectrometry ICP-MS, capillary electrophoresis with laser-induced fluorescence detection, aptamers, ELISA, and immunosensing methods. The HPLC method has become one of the most important techniques to determine mycotoxins in foods as it allows the differentiation of numerous OTA metabolites [1,2,10,11]; still, immunoenzymatic methods can be fast, equally sensitive and reproducible, and less technically and financially demanding [1,2,10]. 

Our data are consistent with numerous other findings published in the literature regarding the OTA concentrations, but the percentage of positive samples is elevated as compared to most of the studies. It is already known that in colostrum OTA levels are increased compared to mature milk (milk/plasma ratio 0.4 versus 0.2) [36].

Biasucci et al. (2011) analyzed the presence of OTA in 57 human milk samples from Italy; the authors reported the presence of OTA in 45 (78.9%), from all samples analyzed [37]. OTA concentrations for positive samples varied between 1.1–75.1 ng/L; the OTA level was lower than 5 ng/L in 26 (58%) of the 45 positive samples. No significant difference was noticed between the OTA concentrations from samples coming from women of Italian origin and foreign nationals residing in Italy. Micco et al. 1995, Italy (27) obtained different results, reporting an OTA concentration in human milk samples which varies between 100 and 12,000 ng/L [38]. Furthermore, in Italy, Turconi et al. (2004) reported OTA concentrations ranging from 1 to 57 ng/L in 86% positive samples out of 231 analyzed [39]. A small percentage of OTA-contaminated samples (1.55%) was determined by Valitutti et al. (2018) in breastfeeding mothers with celiac disease (17–123 ng/L) [40]. The studies conducted by Breitholtz-Emanuelsson et al. (in Sweden, 1993) reported the presence of OTA in 58% of the samples [41]. One of the lowest values for Europe was identified in Switzerland where 10% of samples contained OTA with a maximum value of 14 ng/L [42]. Postupolski et al. (2006) identified OTA in 38% of probes from Poland with an upper limit of 17 ng/L [43]. A Spanish study (2014), as well as a study from Brazil (Andrade et al., 2013), did not detect OTA in human breast milk [44].

Even mycotoxin contamination of food is known to be higher in developed countries; a similar situation was also described in Germany (2013) where 50% of breast milk samples were positive for OTA. It is worth mentioning that there were regional differences regarding OTA levels (Dortmund: 24.4 ± 21.1 ng/L, range: <10–100 ng/L versus Hannover: 14.4 ± 15.1 ng/L, range: <10–78 ng/L) and infant Tolerable Daily Intake (TDI) of 3 ng/kg body weight/day was overcome in almost one-third of the cases [45]. In another study conducted in Slovakia by Dostal et al. (2008), the presence of OTA was confirmed in 23 samples (30.2%) from a total of 76 analyzed with mycotoxin values ranging from 2.3 to 60.3 ng/L [46].

Skaug et al. (2001) highlighted the presence of OTA in 17 of the 80 human milk samples analyzed in Norway; the OTA contamination levels were 10–182 ng/L [47].

In Hungary, Kovacs et al. (1995) analyzed 92 samples of human milk, among which 38 (41.3%) were contaminated with OTA; the determined values ranged from 200 to 7300 ng/L [48].

In some countries, OTA milk concentrations were found to be more than 100-fold higher in comparison with Europe [2]. Studies conducted in Sierra Leone suggested that the high mortality rate in children could be correlated with the increased incidence of OTA in human milk (200 up to 337,000 ng/L) [49]. Increased OTA levels were determined by Gurbay et al. (2009) in Turkey (all samples were positive with values ranging 620–13,111 ng/L) [50]. In the south of Iran, the average concentration of OTA in the samples was 1990 ± 1340 ng/L [6]. In a study carried out in Rabat, Morocco (2020), OTA surpassed 500 ng/L in half of breast milk samples (highest concentration of 10,040 ng/L) and maximum OTA daily intake was exceeded in approximately half of the newborns [51]. Comparative studies from South America indicated that the majority of Chilean samples were OTA-contaminated (the average concentration was 106 ng/L) and 44% of samples from Bolivia contained OTA (20.2–146.1 ng/L) [44]. In Brazil (2021), the values of probable daily intake (PDI) of OTA in lactating mothers based on urinary biomarkers were higher than the upper accepted limit [52]. Seasonal variations regarding OTA levels in breast milk were reported in Turkey and Bolivia [44,53]. 

OTA presence in human breast milk is correlated with a maternal dietary regimen with more than 90 kinds of foodstuffs being involved (preserved meat, cheese, grains, figs, licorice, seeds, tea, black and cayenne pepper, chili powder, etc.) [2,11]. Besides breast milk, rice, barley, oats, and wheat as ingredients of baby food are a significant OTA source for the first years of life [54]. 

The EU Regulation 1881/2006 is still in use even though it has been revised 26 times. Since 2016, this regulation has imposed the rules regarding OTA concentrations in foods for all EU Member States. For example, processed cereal-based foods and baby foods for infants and young children and dietary foods intended specifically for infants have a maximum level of 0.50 ng/g [2]. Generally, OTA contamination levels are increased in countries with a lower economical status but raised concentrations can be present in individual batches in all countries. One of the highest OTA levels reported was 80 mg/kg OTA in moldy bread intended for animal feeding and 20,000 ng/g in pumpkin seeds from China (determination made in Finland, 2015) [2,10].

To date, variable OTA daily limits are set worldwide as follows: provisional tolerable weekly intake (PTWI) at 120 ng/kg bw/week (European Food Authority EFSA—2006), PTWI at 100 ng/kg bw/week (Joint FAO/WHO Expert Committee on Food Additives JEFCA 2007), negligible cancer risk intake (NCRI) at 3–4 ng/kg bw/day (Health Canada, 2010), provisional tolerable daily intake PTDI at 5 ng/kg bw/day (Scientific Committee of Food SCF of the EU) [55]. 

Considering the OTA values found in our study (97.92 ± 64.53 ng/L), and assuming the ingestion of 400–500 mL per day in the first days of life and a body weight of 3.5 kg of the newborn, the OTA quantity surpassed the acceptable lower daily intake (the Daily Intake, TDI), calculated by Kuiper-Goodman and Scott (1989) (0.2 ng–4.2/kg bw) [56]. Other studies also drew attention to the fact that children are most exposed to excess NCRI values due to their lower body weight [57].

In May 2020, the EFSA updated their 2006 opinion regarding the presence of OTA in food. Their conclusion, based on scientific literature between 2006 and 2020, was that the chronic dietary exposure to OTA was between 1.7 and 5.6 ng/kg body weight per day for infants with an average milk consumption between 2.6 and 8.5 ng/kg bw per day for infants with high milk consumption, indicating a possible health concern in this age group [11].

As EFSA indicated, trustworthy information regarding OTA levels in human breast milk is necessary as biomonitoring OTA levels represent a safety measure. Although Romania was one of the 11 countries that established limits on OTA in 1991, data regarding OTA contamination are missing and, to our knowledge, this is the first study reporting OTA levels in breast milk in the Romanian population [2,11].

Generally, cow’s milk is not considered to be an important source of OTA as this mycotoxin can be hydrolyzed by the microflora and by the rumen pH into a less toxic metabolite (OTα) [58]. A 2002 report showed that 7.9% of milk samples were OTA positive in Norway and 13.8% in Sweden [59]. OTA concentrations less than 0.0841 μg/kg were also identified in samples from dairy farms in Beijing, China (2012) [60]. In Nigeria, no OTA presence was identified in cow’s milk (23 samples) but the other seven mycotoxins including aflatoxin M1 contaminated all cow’s milk (max 81 ng/L) [61]. As fungal metabolites survive pasteurization, OTA may be present in dairy products such as cheese and yogurt [62]. Recent data (2021) indicated OTA’s contamination of grated hard cheese (1.3–22.4 µg/kg) [63].

## 4. Conclusions

The performance parameters of the direct competitive immunoenzymatic method, in solid-phase, is recommended for application in determining OTA in biological liquids in which the mycotoxin is found in very small quantities. The linearity of the method, demonstrated on very small analyte concentrations, the detection limit, as well as the limit of quantification, are parameters that place the method in the category of those recommended for the determination of micropollutants in foods, including biological liquids. The results we obtained in determining OTA in colostrum and cow’s milk, as well as the data from the literature, can be a warning sign for the safety of feeding newborn children. This method can now be used to perform a scaled-up version of this study to determine information about the OTA levels in the Romanian population. 

## 5. Materials and Methods

Twelve breast milk (colostrum) samples obtained from mothers admitted at the “Elena Doamna” Maternity Hospital from Iasi, Romania were analyzed. The milk was harvested on the third day from birth, after obtaining informed consent from all the subjects before admission to the study. This study was conducted in accordance with the Declaration of Helsinki and approved by the Ethics Committee of the Faculty of Medicine and Pharmacy, Iași, Romania (24 January 2012). Moreover, fifteen cow’s milk samples (S) were obtained from individual producers from Targu-Frumos county (S1—Boureni; S2—Ion Neculce; S3, S4, S5—Braesti; S6, S7 Targu Frumos; S9, S10—Cristesti; S11—Sacaresti; S12, S13—Prigoreni; S14—Strunga; S15—Buznea).

### 5.1. Equipment

The sample analysis was conducted using an ELISA reader model STAT FAX 303 Plus, manufactured by Awareness Technology, Inc., Palm City, FL, USA.

### 5.2. Reagents

All reagents were multi-analyte and were provided in a compact kit produced by Demeditec Diagnostics GmbH, code DE991OCH01MS, Germany. All samples were assayed in duplicate on ELISA plates.

1. Calibrators with OTA concentrations between 0.0–0.40 ng/mL: each kit contains multi-analyte calibrators with values attributed through a referential method that certifies that the values of the said analytes were established in accordance with WHO-certified reference materials for 6 consecutive levels of concentration, in a liquid conditioning medium, which can be used as such (6 bottles, 1.5 mL each, with OTA concentrations of 0.0, 0.02, 0.05, 0.1, 0.2, 0.4 ng/mL diluted in 70% methanol).
2. Reagent kit for the quantitative detection of OTA, contained all the necessary items that enable the ELISA reaction for the detection of OTA: reactive diluent, enzyme conjugate containing OTA conjugated with horseradish peroxidase (HRP), an underlayer reagent containing stabilized tetramethylbenzidine (TMB), acid stop solution and washing solution as a lyophilized powder from phosphate-buffered saline (PBS) with Tween^®^20 (10 mM phosphate, 137 mM NaCl, 2.7 mM KCl, 0.05% Tween^®^20, pH 7.4); one sachet to be diluted in 1000 mL distilled water. Absolute methanol was purchased from Chemical Company, Romania.

### 5.3. Statistical Analysis

The statistical evaluation was performed using regression analysis, Student’s *t*-test, and ANOVA test. D’Agostino-Pearson, Kolmogorov-Smirnov, and Mann-Whitney rank tests were also applied (MedCalc software). A value of *p* < 0.05 was considered significant.

### 5.4. Sample Preparation

OTA determination in this category of samples requires a 1:3 absolute methanol dilution—750 μL of absolute methanol was added for each 250 μL of the sample (breast milk/cow’s milk). Then, the methanol diluted samples were homogenized through energetic shaking, left to rest at room temperature for 5 min, then centrifuged for 5 min at 3500 rpm. The supernatant was used to determine OTA through the validated direct immunoenzymatic method. 

### 5.5. Method

The quantitative detection of OTA in milk used a direct, competitive, solid-phase immunoenzymatic method. 

In order to begin determination, all reagents were brought to room temperature. 

The sequence of the working stages in the determination of OTA in milk samples implied progressive dilutions of the milk and successive incubations as described in Table 8. 

The procedure was performed in accordance with the test manufacturer’s instructions.

## Figures and Tables

**Table 1:** Method performance parameters.

Tested Sample	%A_0_ Standard 0.02 ng/mL	%A_0_ Sample	%A_0_ Standard < 2 SD	Coefficient of Variation %	ng/mL
Colostrum	86.5	92.3	89.3	1.6	<0.08
Cow’s milk	85.9	91.1	88.9	1.2	<0.08

**Table 2:** OTA calibration curve parameters.

Nr.	Standard	Concentration (ng/mL)	Absorbance	r	Intercept	Slope
1	STD 1	0.4	1.4800	−0.9838	2.824	−3.3430
2	STD 2	0.2	2.0060			
3	STD 3	0.1	2.6070			
4	STD 4	0.05	2.7220			
5	STD 5	0.02	2.7410			
6	STD 6	0	2.7520			

**Table 3:** Statistical parameters of the OTA calibration curve.

Regression Statistics	
Multiple R	0.9838
R Square	0.9681
Adjusted R Square	0.9601
Standard Error	0.1052
Observations	6

**Table 4:** Statistical parameters of the OTA calibration curve.

ANOVA Statistical Analysis					
	df	SS	MS	F	Significance F
Regression	1	1.3426	1.3426	121.2284	0.0004
Residual	4	0.0443	0.0111		
Total	5	1.3869			

**Table 5:** Statistical parameters of the OTA calibration curve.

	Coefficients	Standard Error	t Stat	*p*-Value	Lower	Upper
					95%	95%
Intercept	2.8249	0.0587	48.1313	1.11 × 10^−6^	2.6620	2.9879
Concentration (ng/mL)	−3.4306	0.3116	−11.0104	0.0004	−4.2957	−2.5655

**Table 6:** Limit of detection and limit of quantification for OTA determination in milk samples.

Sample Type	Mean Concentration (ng/mL, *n* = 20)	Standard Deviation (SD)	Limit of Detection (ng/mL)	Limit of Quantification (ng/mL)
Milk	0.0079	0.0039	0.0197	0.0474

**Table 7:** The percentage of recovery.

Tested Sample	Recovery Series I (%)	Recovery Series II (%)	Recovery Series III (%)	Recovery Mean (%)
Human milk	96	110	95	100
Cow’s milk	114	116	113	114

**Table 8:** Workflow in the determination of OTA.

Procedure:	Blank	STD 1	STD 2	STD 3	STD 4	STD 5	STD 6	Sample
		(0.4 ng/mL)	(0.2 ng/mL)	(0.1 ng/mL)	(0.05 ng/mL)	(0.02 ng/mL)	(0 ng/mL)	
a. Addition	x	200 μL of reactive diluent was added to each well						
b. Addition	x	100 μL	100 μL	100 μL	100 μL	100 μL	100 μL	100 μL
c. Homogenization	x	2–3 successive pipetting for homogenization were performed.						
d. Transfer	x	100 μL of contents from each mixing well of microtiter plate was transferred.						
		to corresponding Anti OTA antibody coated well from reaction microplate.						
e. Incubation	x	30 min at ambient temperature (22 °C).						
f. Wash	x	The content of the wells was removed and then wells were washed 3 times						
		with washing buffer PBS-Tween (pause between washes was 60 s).						
		After the last wash the excess moisture was absorbed by blotting on filter paper.						
g. Addition	x	100 μL OTA-horseradish peroxidase (HRP) conjugate was added to each well.						
h. Incubation	x	30 min at ambient temperature (22 °C).						
i. Wash	x	Three successive washes were carried out with washing buffer PBS-Tween.						
		After the last wash the excess moisture was absorbed by blotting on filter paper.						
j. Addition	x	100 μL substrate solution TMB was added to each well. Being a chromogenic						
		substrate for HRP, TMP produced a deep blue color during the enzymatic						
		degradation of hydrogen peroxide by HRP.						
k. Incubation	x	10 min at ambient temperature (22 °C).						
l. Homogenization	x	2–3 successive pipetting for homogenization were performed.						
m. Reading	x	Using a spectrophotometer, optical density was read at λ = 450 nm with 630						
		nm reference filter. The intensity of the color was directly proportional with the						
		quantity of linked conjugate and indirectly proportional to the quantity of OTA						
		present in the standard or in the sample.						

## Supplementary Materials

The following are available online at https://www.mdpi.com/article/10.3390/toxins13100673/s1, Table S1: Demographic data, Table S2: Nutrition-related data.
