# Supplementary material for: An Immunoenzymatic Method for the Determination of Ochratoxin A in Biological Liquids (Colostrum and Cow’s Milk)
Source: Toxins (Basel). 2021 Sep 22;13(10):673. doi: 10.3390/toxins13100673 (PMC8538136; doi:10.3390/toxins13100673)
Supplement: Supplementary file 1 [file toxins-13-00673-s001.zip › toxins-1378779-supplementary.pdf]

Supplementary Materials: An Immunoenzymatic Method for the Determination of Ochratoxin A in Biological Liquids (Colostrum and Cow's Milk)

Magdalena Cuciureanu, Cristina Tuchiluș, Anca Vartolomei, Bogdan Ionel Tamba and Lorena Filip

Table S1. Demographic data.

Subject	Age	Place of Residence	Number of Children	Higher Education (Faculty)	Height (m)	BW* (kg)	BMI** (kg/m2)
1	25	town	1st child	yes	1.67	65	23.307
2	34	town	2nd child	yes	1.58	65	26.037
3	19	village	1st child	no	1.75	70	22.857
4	22	village	1st child	no	1.62	80	30.483
5	40	town	3rd child	no	1.65	78	28.65
6	37	town	1st child	yes	1.58	63	25.236
7	32	village	2nd child	yes	1.7	80	27.682
8	22	town	2nd child	no	1.73	91	30.405
9	27	village	3rd child	no	1.62	79	30.102
10	35	town	2nd child	yes	1.66	78	28.306
11	29	town	2nd chid	no	1.58	72	28.842
12	29	village	2nd child	no	1.62	71	27.054

*body weight (kg), **body mass index = BW (kg)/Height $(m)^2$

Table S2. Nutrition-related data.

Subject	Servings of Nuts/Week	Servings Meat/Week	Predominant Source of Food: Own Farm/Local Market/Supermarket	Servings of Milk and Dairy Products/Week	Servings of Fresh Fruits/Week	Frequent Consumption of Dried Fruits (Almost Each Day)
1	7	0	supermarket	5	7	yes
2	7	2	supermarket	7	7	yes
3	3	4	own	7	4	no
4	8	3	own	6	7	no
5	4	6	supermarket	4	5	no
6	5	2	local market	6	3	no
7	7	7	own	5	3	yes
8	8	6	local market	7	5	yes
9	7	5	local market	7	4	no
10	7	5	supermarket	4	3	no
11	5	2	supermarket	5	6	yes
12	5	3	local market	7	5	no
